# MANAGEMENT OF VARICEAL HEMORRHAGE: CURRENT CONCEPTS

**DOI:** 10.1590/S0102-67202014000200011

**Published:** 2014

**Authors:** Fabricio Ferreira COELHO, Marcos Vinícius PERINI, Jaime Arthur Pirola KRUGER, Gilton Marques FONSECA, Raphael Leonardo Cunha de ARAÚJO, Fábio Ferrari MAKDISSI, Renato Micelli LUPINACCI, Paulo HERMAN

**Affiliations:** 1 Serviço de Cirurgia do Fígado e Hipertensão Portal, Departamento de Gastroenterologia, Hospital das Clínicas da Faculdade de Medicina da Universidade de São Paulo (1Liver Surgery Unit, Department of Gastroenterology, University of São Paulo Medical School; 2 Serviço de Transplantes, Departamento de Cirurgia, Santa Casa de Misericórdia (2Transplant Service, Department of Surgery, Santa Casa de Misericórdia de São Paulo); 3 Instituto do Câncer do Estado de São Paulo in São Paulo, SP, Brazil; (3Instituto do Câncer do Estado de São Paulo in São Paulo, Brazil); 4 Service de Chirurgie Générale, Viscérale et Endocrinienne, Hôpital Pitié Salpetrière in Paris, França (4Service de Chirurgie Générale, Viscérale et Endocrinienne, Hôpital Pitié Salpetrière in Paris, France)

**Keywords:** Portal hypertension, Esophageal and gastric varices, Liver cirrhosis, Schistosomiasis mansoni

## Abstract

**Introduction:**

The treatment of portal hypertension is complex and the the best strategy depends on the underlying disease (cirrhosis vs. schistosomiasis), patient's clinical condition and time on it is performed (during an acute episode of variceal bleeding or electively, as pre-primary, primary or secondary prophylaxis). With the advent of new pharmacological options and technical development of endoscopy and interventional radiology treatment of portal hypertension has changed in recent decades.

**Aim:**

To review the strategies employed in elective and emergency treatment of variceal bleeding in cirrhotic and schistosomotic patients.

**Methods:**

Survey of publications in PubMed, Embase, Lilacs, SciELO and Cochrane databases through June 2013, using the headings: portal hypertension, esophageal and gastric varices, variceal bleeding, liver cirrhosis, schistosomiasis mansoni, surgical treatment, pharmacological treatment, secondary prophylaxis, primary prophylaxis, pre-primary prophylaxis.

**Conclusion:**

Pre-primary prophylaxis doesn't have specific treatment strategies; the best recommendation is treatment of the underlying disease. Primary prophylaxis should be performed in cirrhotic patients with beta-blockers or endoscopic variceal ligation. There is controversy regarding the effectiveness of primary prophylaxis in patients with schistosomiasis; when indicated, it is done with beta-blockers or endoscopic therapy in high-risk varices. Treatment of acute variceal bleeding is systematized in the literature, combination of vasoconstrictor drugs and endoscopic therapy, provided significant decline in mortality over the last decades. TIPS and surgical treatment are options as rescue therapy. Secondary prophylaxis plays a fundamental role in the reduction of recurrent bleeding, the best option in cirrhotic patients is the combination of pharmacological therapy with beta-blockers and endoscopic band ligation. TIPS or surgical treatment, are options for controlling rebleeding on failure of secondary prophylaxis. Despite the increasing evidence of the effectiveness of pharmacological and endoscopic treatment in schistosomotic patients, surgical therapy still plays an important role in secondary prophylaxis.

## Abstract (pt)

**Introdução:**

O tratamento da hipertensão portal é complexo e a definição da melhor estratégia depende da causa subjacente (cirrose vs. Esquistossomose), da condição clínica e do momento em que é realizado (episódio agudo de hemorragia ou como profilaxia pré-primária, primária ou secundária). Com o advento de novas opções medicamentosas e o desenvolvimento da endoscopia e radiologia intervencionista, o tratamento da hipertensão portal tem sofrido grande transformação nas últimas décadas.

**Objetivo:**

Avaliar os avanços e as estratégias empregadas no tratamento emergencial e eletivo da hemorragia varicosa em pacientes cirróticos e esquistossomóticos.

**Método:**

Revisão bibliográfica nas bases de dados PubMed, Embase, Lilacs, SciELO e Cochrane até junho de 2013, com os descritores: portal hypertension, esophageal and gastric varices, variceal bleeding, liver cirrhosis, schistosomiasis mansoni, surgical treatment, pharmacological treatment, secondary prophylaxis, primary prophylaxis, pré-primary prophylaxis.

**Conclusão:**

Com relação à profilaxia pré-primária não existem estratégias específicas; a melhor recomendação é tratamento da doença de base. A proflaxia primária em pacientes cirróticos deve ser feita com betabloqueadores ou terapêutica endoscópica com ligadura elástica. Existe controvérsia quanto à efetividade da profilaxia primária em pacientes esquistossomóticos; quando indicada, faz-se com betabloqueadores ou terapêutica endoscópica nas varizes de maior risco. O tratamento do sangramento agudo é o com melhor sistematização e mais alto nível de evidência; a associação de drogas vasoconstritoras e terapia endoscópica proporcionou queda significativa na mortalidade nas últimas décadas. O TIPS e o tratamento cirúrgico são opções na terapia de resgate. A profilaxia secundária é feita com terapia farmacológica (betabloqueadores) e endoscópica em pacientes cirróticos. Na falha da profilaxia secundária, o TIPS ou o tratamento cirúrgico são opções viáveis para controle da recidiva hemorrágica. Apesar do aumento das evidências da eficácia da terapêutica farmacológica e endoscópica em pacientes esquistossomóticos, o tratamento cirúrgico ainda tem papel preponderante na profilaxia secundária destes pacientes.

## INTRODUCTION

The portal system is a venous plexus within low pressure, physiologically less than 5 mmHg. Portal hypertension (PH) represents a clinical syndrome characterized by a sustained increase in venous pressure at levels over than physiological. It becomes clinically relevant when values overtake 10 mmHg increasing the risk of emergence of esophagogastric varices (EGV). Portal vein pressure higher than 12 mmHg increase the risk of EGV rupture^13,21^.

Elevated portal vein flow causing directly PH is a rare event and usually is represented by arterial-portal fistulas with congenital, traumatic or neoplasic origins. The increased resistance is the most common initial pathophysiological condition and can be classified as pre-hepatic, intra-hepatic or post-hepatic. The intra-hepatic conditions are classified according to the location of structural damage in the liver parenchyma as pre-sinusoidal (i.e. hepatosplenic form of schitossomiasis [HSS] and congenital liver fibrosis), sinusoidal (i.e. cirrhosis) and pos-sinusoidal (i.e. veno-occlusive disease). In our population, the most of PH cases is due HSS and liver cirrhosis.

The treatment of PH depends on the underlying disease; patient's clinical condition and time on it is performed. Patients with bad liver function have different approach than patients with normal liver function, as patients with HSS. Moreover, the treatment can be emergency (acute bleeding) or elective as pre-primary, primary or secondary prophylaxis. Therefore, there is no single modality of treatment for all of these conditions.

The aim of this review is to analyze recent improvements and strategies used in the emergency and elective treatments of EGV bleeding in patients with liver cirrhosis and HSS.

## METHODS

A literature review was performed based on search in PubMed, Embase, Lilacs, SciELO and Cochrane databases until June 2013. The keywords used were portal hypertension, esophageal and gastric varices, variceal bleeding, liver cirrhosis, schistosomiasis mansoni, surgical treatment, pharmacological treatment, secondary prophylaxis, primary prophylaxis, pre-primary prophylaxis.

### Portal hypertension treatment

The prevalence of EGV in cirrhotic patients varies according to the liver function. The EGV are present in 30% of patients with compensated liver function (Child A), while in patients without compensated liver function (Child B and C) can be present EGV in 60% of cases^2^. Moreover, EGV can progress from small to large caliber according to clinical worsening of the cirrhosis. Merli et al.^29^ observed progression of caliber in 12% of cirrhotic patients at one year and 31% at three years. Thus, the endoscopic screening of EGV is recommended in all cirrhotic patients. Child A patients without EGV might undergo endoscopy investigation each two or three years, Child B and C patients might undergo endoscopy control annually^2,13^.

### Pre-primary prophylaxis

Pre-primary prophylaxis intends measures to avoid EGV rising in patients with PH. There is no evidence of benefits in patients with HSS. The current recommendations come from data of cirrhotic patients and they did not demonstrate benefits of using non-selective beta-blockers (NSBB) for this group of patients. The most effective action as pre-prophylaxis is the treatment of the underlying disease (hepatitis B, hepatitis C, autoimmune hepatitis, etc.) that could slow down the progression of the liver disease. In patients with HSS, the treatment with anti-helminthic drugs (oxamniquine and praziquantel) reduces the parasitic load and can avoid the progress of fibrotic hepatic disease^29^.

### Primary prophylaxis

Conceptually, employs measures to minimize the risk of first hemorrhage in patients with PH and presence of EGV.

#### Cirrhotic patients

The lifetime risk of bleeding ranges from 20 to 40%, being directly related to variceal caliber, presence of red spots, hepatic venous pressure gradient ≥12 mmHg and severity of liver disease. Patients with compensated liver function present risk of bleeding in around 4% per year opposing 7.6% for patients with decompensated liver function^8^.

The strategies that have being effective in the primary prophylaxis in cirrhotic patients were NSBB (propranol and nadolol are the most used) and endoscopic therapy, especially band ligation (BL). Actually, surgery is rarely used as primary prophylaxis in patients with liver cirrhosis^30^. Primary prophylaxis is indicated in cirrhotic patients based on variceal caliber and the presence of other risk factors for rupture.

#### Patients with small EGV

Child A patients, without any other risk factor for bleeding, can be benefited by NSBB to avoid bleeding (individualized use). In this group of patients, the use of NSBB reduces the progression of varices in three years (11% vs 37% in placebo group) and the risk of bleeding in five years (12% vs 22% in the placebo group)^28^. Patients without compensated liver function (Child B and C) have higher risk of bleeding and might receive NSBB as primary prophylaxis.

#### Patients with medium and large EGV

This group of patients should undergone primary prophylaxis independently of liver function and other risk factors for bleeding on endoscopy evaluation. For this group of patients, prophylaxis can be offered based on NSBB or BL. Endoscopic sclerotherapy presents controversial results and higher risk of complications; subsequently it has been proscribed as primary prophylaxis in cirrhotic patients^19^. There are many systematic reviews comparing BL and NSBB, both in primary prophylaxis. Gluud et al.^20^ demonstrated better control of first bleeding in patients underwent BL, without differences in morbidity and mortality. However, this superiority was not verified when just studies with adequate randomization were included. Afterwards, both modalities can be used as primary prophylaxis of EGV bleeding in cirrhotic patients with medium and large EGV. The Baveno V consensus recommended that the decision about the use of each modality should be made based on local resources, team experience and individual necessity of each patient^13^.

#### HSS patients

There are few data about primary prophylaxis for patients with HSS. Currently surgical therapy is rarely indicated in these patients^31,33^. The prophylaxis for this group of patients remains controversial since there is no evidence of future bleeding based on only presence of EGV. In general, these patients have a risk of bleeding in 11-30%, with mortality reaching 11-20%^17,26^. In this context, the primary prophylaxis would be indicated just for patients with high risk of bleeding with larger varices and endoscopic signs of risk.

The NSBB use in this group was debated since it demands high doses with many collateral effects. Recently, new evidences indicated that its use is effective for patients who never presented bleeding, with significant decrease of pressure in esophageal varices^15^. Some authors demonstrated good results with endoscopic treatment to control EGV in patients with HSS. Thus, when indicate for this group, primary prophylaxis should be done with NSBB or BL for patients who present higher-risk varices.

### Acute hemorrhage

**Figure 1:**
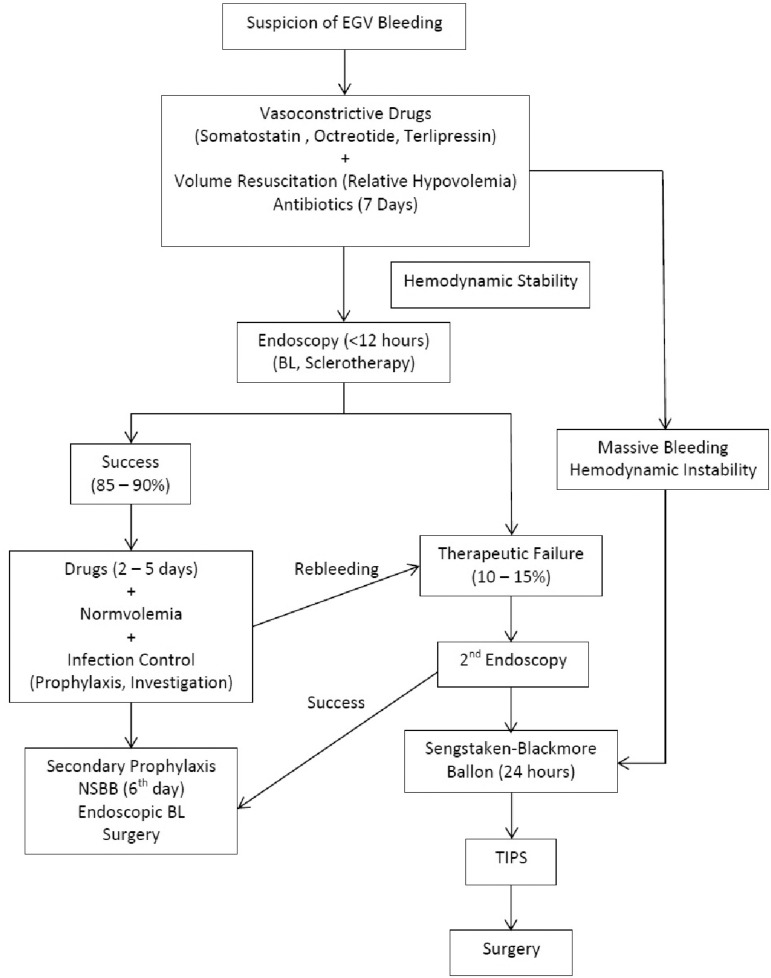
Flowchart of management of acute variceal hemorrhage from Liver Surgery Unit of University of São Paulo Medical School EGV=Esophagogastric varices; BL=band ligation; TIPS=transjugular intrahepatic portosystemic shunt

The bleeding caused by EGV rupture is a medical emergency with high mortality, so its management has to be done in an intensive care unit. The mortality in the first episode is directly related to patients' liver function and clinical condition; it can reach 10-20% in patients with HSS^17,26^ and 30-45% in cirrhotic patients. In the last decades, while standardized approach to patients with acute variceal hemorrhage, the mortality has decreased from 43% to 14%^4^. The flowchart proposed by Liver Surgery Unit of University of São Paulo Medical School is demonstrated in Figure 1.

The key points of acute variceal bleeding management are: volume infusion, pharmacological and endoscopic control of hemorrhage and infection prophylaxis.

During active bleeding, the volume resuscitation should be undertaken promptly with the goal of restore blood pressure and perfusion, but it might be done carefully to avoid overload volume which could increase portal vein pressure and subsequently the risk of rebleeding by EGV. Actually, the use of saline solution and blood transfusion have been administrated during acute bleeding looking for hemodynamic stability keeping systolic arterial pressure around 90-100 mmHg, cardiac frequency <100 beats/min and hemoglobin level in 7-9 g/dl (hematocrit in 21-27%)^2,19^.

The better results to control acute bleeding with more than 90% of succeed interventions are obtained combining endoscopy therapy and drugs that decrease the splanchnic blood flow as somatostatin, octeotride and terlipressin^9,13^.

The pharmacological treatment has efficacy in the control of acute bleeding and to avoid rebleeding, it should to be use in variceal bleeding suspicion, even before of endoscopy approach. The only drug associated with decrease of mortality was terlipressin (decreasing the risk in 34%), thus, it is the best choice to treat EGV bleeding^23^. Terlipressin is a synthetic analog of vasopressin with less collateral effects and longer half-life than it. The initial dose is 2 mg followed by 1-2 mg every 4 h (adjusted by weight : <50 Kg 1 mg; 50-70 Kg 1.5mg; >70 kg 2 mg) during 2-5 days.

The endoscopy is mandatory and should be done as soon as possible in EGV bleeding, just after hemodynamic management, in the first 12 h after patient admission. The BL is the preferable modality since it has effective bleeding control in 86-92% of cases. When BL was compared to sclerotherapy, it presented lower risk of rebleeding, lower frequency of adverse effects, lower number of sessions to obliterated EGV and better overall survival^25^. Therefore, BL should be treatment of choice, but sclerotherapy is also acceptable when BL is not possible.

Antibiotic prophylaxis has an important role in the treatment of digestive bleeding in cirrhotic patients and it should be initiated as soon as bleeding episode appears. Infection is identified in 25-50% of patients in their admission or during the length of stay for EGV bleeding, being commonly associated to spontaneous bacterial peritonitis, urinary tract infection and pneumonia. The presence of infection is an independent predictor of rebleeding and mortality. In a recently published meta-analysis, Chavez-Tapia et al.^5^ demonstrated that antibiotics can reduce bacterial infection in 64%, rebleeding rates in 47% and mortality related to bleeding in 21%. Oral quinolone (especially norfloxacin 400 mg twice a day, during seven days) are the choice of treatment. Endovenous quinolones (i.e. ciprofloxacin) are used when oral administration is not feasible. Looking for high-risk patients (presence of ascites, encephalopathy, jaundice and malnutrition), there is evidence that endovenous ceftriaxone 1 g per day can be more effective to control infection. There is neither evidence nor recommendation of antibiotic use as infection prophylaxis in patients with HSS.

#### Therapeutic failure

It is define as death or necessary changing of therapy after first five days post-hematemesis or pharmacological aspiration in nasogastric tube more than 100 ml in two or more hours after the beginning of specific and endoscopic therapy; hemodynamic shock or decreasing of 3 g/dl of hemoglobin level (9% in hematocrit level) in 24 h without blood transfusion^13^. The therapeutic failure occurs after standard treatment in 10-15% of cases^6^, more often related to patients with decompensated liver disease, coursing with mortality between 30-50%. The main related risk factors to therapeutic failure are Child C, MELD>18, active bleeding during endoscopy and hepatic vein pressure gradient ≥20 mmHg^18^.

Approaching therapeutic failure, vasoconstrictive drugs can be used in maximal dose, a second attempt by endoscopy can be done as well, and in presence of massive bleeding, insufflation of esophageal balloon (the most use is Sengstaken-Blakemore) might be done (Figure 1). Its efficacy in bleeding control is around 80-90%; however, rebleeding rate is high (over 50%). This balloon can be kept insufflated no more than 24 h since there is risk of ischemic esophageal lesions, so it is just considered as bridge treatment.

Considering failure of endoscopic therapy and maximal doses of pharmacological treatment, the use Transjugular Intrahepatic Portosystemic Shunt (TIPS) can be done to decrease PH, subsequently decreasing pressure in EGV. The main advantage of TIPS comparing to surgical shunts are lower morbidity and mortality rates. TIPS is already well established as salvage treatment in cirrhotic patients controlling bleeding in over 90% of cases and with rebleeding rate in 12%^18^. Concerning patients with high-risk therapeutic failure (Child C, Child B presenting active bleeding and hepatic vein pressure gradient ≥20 mmHg), the early use of TIPS, in the first 72 h, were associated with better results to bleeding control and mortality than endoscopic and pharmacological therapy^18^. The main concerns of its use are high rates of stenosis (over 50%) demanding new interventions, moreover, it presents high rate of encephalopathy (20-40%)^6,31^. There is lack of experience of this technique with HSS patients, but it could be an option in refractory cases or in patients without clinical performance to undergo surgery.

#### Surgical treatment

Currently, the surgical treatment is reserved for patients who are refractory to clinical, endoscopic and endovascular treatment^30^. There is no evidence about the best surgical treatment to be used. The treatment of choice has to be done based on patient's clinical condition, local resources, surgeon expertise, looking always for bleeding control with shorter procedure as possible, especially in patients with hemodynamic instability.

Non-selective derivations are the most used procedures in cirrhotic patients since its technical feasibility and early decreasing in portal pressure. Although some centers related acceptable results using portocaval derivation, the most of centers related high mortality (40-50%) and high rate of encephalopathy (around 40%)^3^. The most used shunt as salvage therapy are portosystemic calibrated shunt (portocaval and mesocaval), through interposition of vein graft or polytetrafluoroethylene prosthesis with 8-10 mm. Calibrated shunts provide control of EGV bleeding with less encephalopathy and liver damage in long term results.

Azygoportal disconnection has more restrict use; however, it is considered an option for patients who are not candidates for derivations, as patients with portal vein thrombosis or patients in not specialized centers. Several disconnection procedures have been proposed as treatment of EGV bleeding, many of them became well accepted for relative technical facility, being valid option as salvage therapy. Among disconnection procedures, the direct ligation of EGV and esophageal transection merits to be cited. Esophageal transection is a simple technique (does not demand specialized expertise) and effective as salvage therapy in severe hemodynamic conditions. It is performed through a vertical gastrotomy on anterior wound and introduction of circular stapler into distal esophagus lumen, and is positioned 1-2 cm above esophagogastric transition zone. The circular stapler is applied splitting and performing anastomosis at same time. The rational of this technique is to disconnect the hepatofugal flow present in PH, since submucosal venous plexus of esophageal is sectioned. So, the blood from portal system does not reach azygo vein through EGV after esophagus transection and anastomosis. This technique can provide bleeding control; however, its isolated use presents high recurrence rates (20-50%)^36^^\^. The better results are obtained when the esophageal transection is associated with esophagogastric devascularization, with good results in bleeding control and long-term outcomes^38^.

In patients with HSS, the options of surgical treatment for bleeding control are more restrict in the literature. But they are also preconized as treatment following failure of pharmacological and endoscopic treatment, without consensus about any standard surgical approach.

The portosystemic calibrated shunts are less used for the adverse effects discussed above (encephalopathy and liver dysfunction), but they can be used in refractory cases without another therapeutic options. The distal splenorenal shunt (Warren operation) is a technically complex surgery and then not often used as salvage therapy^33^.

The azygoportal disconnection procedures can be placed in selected cases and the surgical techniques are the same used for cirrhotic patients. There are experienced services performing surgical ligation of EGV, modality of direct approach to the vessels^22^. There are some options to approach and direct ligate EGV: intra-esophagic, extramucosal and transgastric approaches. Among these options, the transgastric ligation emerged as an option to avoid thoracotomy and esophagotomy, and their inherent morbidity. It was proposed by Crawford et al.^7^ who performed the procedure exclusively by abdominal approach, through gastrotomy and direct ligation of EGV. Ever since, this technique has been used to treat acute bleeding (with good immediate bleeding control, but high rates of rebleeding) and elective cases as well, mainly in cases presenting gastric varices. The advantages of this technique are the relative ease to perform and direct access to gastric varices that are usually challenging in patients with PH. The gastric varices are present in 20% of PH cases, and they rise to 35% in HSS patients^16^. Gastric varices represent less than 2% of initial presentation of digestive bleeding, however their bleeding are more severe than esophageal varices. There is a high mortality even in patients with preserved liver function, varying in 29-55% in HSS patients^16^. Gastric varices are associated with difficulty to endoscopic control, especially those placed in gastric fundus, thus the surgical treatment is justified for refractory cases.

The esophagogastric devascularization can also be applied in treatment of acute bleeding in HSS patients, isolated or combined with other procedures, as esophageal transection. This modality was recommended by Sugiura and Futagawa^36^, and japanese studies showed that esophagastric transection combined with devascularization have presented good results in acute bleeding control mainly in non-cirrhotic PH.

Esophagogastric devascularization and splenectomy (EGDS) is also a valid option for acute bleeding control, however it should be avoid in patients with hemodynamic instability since they could not tolerate the procedure.

### Secondary prophylaxis

It places actions to minimize the risk of rebleeding in patients who already had EGV hemorrhage.

#### Cirrhotic patients

Patients who had a first EGV bleeding have risk of rebleeding in around 60-70% in one year and high mortality rate (33%)^1^. Thus, it is mandatory that all patients who presented acute bleeding and were treated with pharmacological and endoscopic treatment also receive secondary prophylaxis. There is no recommendation in the literature for secondary prophylaxis for patients who underwent TIPS or portosystemic shunt as treatment for acute bleeding.

NSBB, nitrates and endoscopic therapy compose secondary prophylaxis. NSBB are effective preventing rebleeding (decreasing of risk to 40-45%) and it improves long-term outcomes (increasing overall survival in 5 % in two years)^1^. It should be initiated as soon as possible; it should be in sixth day post-hemorrhage and kept continuously since its interruption can cause rebound increasing of portal pressure and predisposing rebleeding. Although its efficacy, only 40% of patients treated with NSBB reach reduction of portal pressure <12 mmHg or at least less 20% of basal level, these patients are considered as responders^2,13^. Nitrates can be added to NSBB for non-responders, since it can cause synergic effect to decrease portal pressure. Several studies showed this pharmacological association decreasing rebleeding rates; however, it presents more collateral effects without impact in overall survival.

Regarding endoscopic therapy, BL is considered better than esclerotherapy to prevent rebleeding and increase survival. A meta-analysis by Laine and Cook^25^ observed reduction of risk of rebleeding in 48% and mortality in 23% in patients who underwent secondary prophylaxis with BL when compared to those who underwent esclerotherapy. Randomized clinical trials comparing pharmacological therapy and BL demonstrated conflicting results that did not demonstrate clear superiority of any of them alone.

The pharmacological therapy with NSBB associated to endoscopic therapy with BL is the best rational presenting better results in secondary prophylaxis. The early administration of NSBB can reduce the risk of bleeding until undergoes endoscopic therapy. Randomized clinical trials demonstrated better results on rebleeding control in patients who received combined therapy than the group of patients that received only endoscopic BL (11-14% vs 27-38%)^14^.

Failure in secondary prophylaxis can be presented in 10-20% of cases, even when adequate treatment is instituted^14^. The salvage therapy options are TIPS and surgical treatment. Several studies have shown TIPS as effective to secondary prophylaxis when compared to endoscopic and pharmacological therapy^6^. A meta-analysis including 12 randomized clinical trials showed better results on rebleeding control in TIPS group when compared to endoscopic therapy group (19% vs 44%, O.R.= 0.32, 95% I.C. 0.24-0.43), however, they presented higher rates of post-treatment encephalopathy (33% vs 19%, O.R.= 2.21, 95% I.C. 1.61-3.03)^40^.

Among surgical options, the liver transplantation could be considered the best of them, since it would treat not only PH but also their baseline disease. However, most patients would not have access to this treatment as secondary prophylaxis. Thus, procedures that would definitely treat EGV have role as salvage therapy. The options most placed are non-selective portosystemic derivations, especially calibrated shunts (portocaval or mesocaval) and selective derivations as distal splenorenal shunt (Warren operation).

Comparatives studies have demonstrated the surgical treatment as effective in secondary prophylaxis, with better bleeding control when compared to endoscopic therapy. A Cochrane meta-analysis reported by Khan et al.^24^ observed that portosystemic shunts decreases rebleeding risk in 86%, and distal splenorenal shunt in 83%, when compared to endoscopic therapy, but without impact in overall survival.

The choice of standard modality (TIPS vs surgical treatment) as salvage therapy for cirrhotic patients who failed on secondary prophylaxis remains controversial. The good results and lower morbidity and mortality have made TIPS first choice for many authors^2,6^. However, subgroups of patients with chronic liver disease have presented favorable results with surgical treatment. Several studies showed superiority of surgery when compared to TIPS in patients with preserved liver function (Child A), with lower rates of rebleeding and reintervention, and better overall survival. Rosemurgy et al.^34^ compared patients underwent portocaval calibrated shunt (8 mm) to patients underwent TIPS and obtained lower rates of rebleeding (7.6% vs 30%) and reinterventions (10.6 vs 48.5%) in the surgical group. Long-terms results were better in patients who were Child A and MELD<14.

In this context, TIPS has been proposed as first choice for patients with decompensated liver function and with perspective liver transplantation (Child B and C, MELD>14). Surgery seems to be the better for patients with compensated liver function (Child A, MELD<14).

#### HSS patients

This group of patients presents high-risk of rebleeding, with rates around 60-75%^22^. Since these patients need high doses of NSBB, they presented more collateral effects, and consequently they also presented lower treatment adherence. Moreover, there is no data supporting its role into rebleeding prevention. Looking for isolated endoscopic therapy, scleroterapy presents relapse of EGV in 60% of cases and rate of reebleding around 30%^35^. The BL seems offer better results and tolerance, however, there still no conclusive results for its isolated efficacy for rebleeding prophylaxis. Therefore, many services indicate surgical treatment as secondary prophylaxis for HSS patients^17,26,31,32^.

The surgical treatment in HSS patients have as target to avoid rebleeding keeping liver function preserved, not inducing encephalopathy, and additionally treating hypersplenism. Many techniques were proposed, but none of them cover all of these premises. In this context, two techniques have been presenting more acceptances for specialized centers: distal splenorenal shunt (Warren operation) and EGDS.

#### Distal splenorenal shunt

This technique was independently proposed by Warren et al.^39^ and Teixeira et al.^37^ in 1967, and it intends to decrease the EGV pressure through deviation of spleen flow to systemic circulation (end-to-side splenorenal anastomosis), maintaining portal vein flow and liver perfusion. So, two distinct flow zones are created, one representing EGV which has the flow decreased by short gastric vessels and spleen, then systemic circulation through left renal vein (low pressure zone); the other one comprise hepatic hilum which has preserved portal flow (high pressure zone).

This technique presents good results with rates of rebleeding in 2.8-7%. However, it is a complex surgery with high mortality around 4-15%. The thrombosis of anastomosis is also common occurring in 15% of cases^32^.

Many patients develop transient postoperative ascites, which is attributed to the manipulation of retroperitoneal lymphatic vessels by dissecting the renal and splenic veins. The pressure difference between the two areas described: territory of varices (low pressure) and portal (high pressure) stimulates the formation of new vessels, which establishes communication between them. Thus, loss of selectivity and development of encephalopathy could be present in up to 15% of cases^32^. Before the procedure should be evaluated the presence of pulmonary hypertension, which can occur in 20% of patients with HSS^12^. By diversion of blood to the systemic circulation there is increased venous return, which may result in acute cardiac overload and consequent failure in patients with pulmonary hypertension. Therefore, before the operation the pressure in pulmonary artery should be measured directly or indirectly, and in patients with pressure >25 mmHg derivation should not be indicated^11^.

#### Esophagogastric devascularization and splenectomy (EGDS)

The azygoportal disconnection intends to interrupt the hepatofugal flow across EGV zone performing devascularization of superior 2/3 of stomach veins and distal esophagus. The liver remains supplied by splanchnic system through portal vein, which avoids late hepatic dysfunction.

Isolated splenectomy was also used as secondary prophylaxis, however has a high failure rate (30-56%) and it was abandoned for the most of services^33^. However, splenectomy is usually combined to azygoportal disconnection techniques. Hemodynamic studies showed that ligation of splenic artery, when associated with azypoportal disconnection, decrease portal vein pressure in 30%, and also improve the hyper dynamic system pattern^10^.

The EGDS has rebleeding rates in 5-16%, and mortality in 1-7%, but without any cases of encephalopathy since there is no blood deviation to systemic circulation^32^. Better results on rebleeding control are reached when endoscopic therapy is applied post-operatively to EGDS^26,35^. In this context, lower morbidity and mortality, absence of post-operative encephalopathy and good rebleeding control made the association of EGDS and post-operative endoscopic therapy as first choice in HSS patients. Makdissi et al.^26^ related 97 patients who underwent both treatment modalities and were followed up five years and more; they present eradication of EGV in 85.6% of cases, with low morbidity and mortality. This study demonstrated that no rebleeding in 20 years was possible in 82.5% of patients with this technique, moreover, also treats hypersplenism.

## CONCLUSION

The PH treatment is complex and the choice of best therapeutic strategy depends on many factors: baseline disease, patient's clinical performance and the timing when it is done (emergency or prophylactic approaches). The levels of evidence in the literature concerning PH treatment are better for cirrhotic patients than HSS patients. New pharmacological options and improvements on endoscopy in the last decades have been providing important progress in PH treatment. Surgical treatment actually has no role as pre-primary or primary prophylaxes. It has being used as salvage therapy in patients with acute hemorrhage and as secondary prophylaxis in cirrhotic patients, still having preponderant role in secondary prophylaxis of HSS patients.
